# Outdoor Formaldehyde and NO_2_ Exposures and Markers of Genotoxicity in Children Living Near Chipboard Industries

**DOI:** 10.1289/ehp.1307259

**Published:** 2014-04-02

**Authors:** Alessandro Marcon, Maria Enrica Fracasso, Pierpaolo Marchetti, Denise Doria, Paolo Girardi, Linda Guarda, Giancarlo Pesce, Vanda Pironi, Paolo Ricci, Roberto de Marco

**Affiliations:** 1 Unit of Epidemiology and Medical Statistics, Department of Public Health and Community Medicine, University of Verona, Verona, Italy; 2 Section of Pharmacology, Department of Public Health and Community Medicine, University of Verona, Verona, Italy; 3 Unit of Epidemiology, National Health Service Mantua, Mantua, Italy

## Abstract

Background: Industrial air pollution is a public health hazard. Previous evidence documented increased respiratory symptoms and hospitalizations in children who live near the factories in the largest chipboard manufacturing district in Italy (Viadana).

Objectives: We evaluated the association of outdoor exposure to formaldehyde and nitrogen dioxide (NO_2_) with markers of early genotoxic damage in oral mucosa cells of randomly selected children (6–12 years of age) living in Viadana.

Methods: In 2010–2011, DNA strand breaks and nuclear abnormalities were evaluated in exfoliated buccal cells by the comet and micronucleus assays, respectively, and formaldehyde and NO_2_ were monitored by passive sampling. Annual exposure estimates to pollutants were assigned to children’s houses by spatial interpolation.

Results: Of 656 children, 413 (63%) participated. Children living near (< 2 km) the chipboard industries had the highest average exposure to formaldehyde and NO_2_ (*p* < 0.001). A 1-SD increase in formaldehyde (0.20 μg/m^3^) was associated with a 0.13% (95% CI: 0.03, 0.22%) higher comet tail intensity, a 0.007 (95% CI: 0.001, 0.012) higher tail moment, and a 12% relative increase [relative risk (RR) = 1.12; 95% CI: 1.02, 1.23] in nuclear buds. A 1-SD NO_2_ increase (2.13 μg/m^3^) was associated with a 0.13% (95% CI: 0.07, 0.19%) increase in binucleated cells and a 16% relative increase (RR = 1.16; 95% CI: 1.06, 1.26) in nuclear buds.

Conclusions: Exposure to pollutants was associated with markers of genotoxicity in exfoliated buccal cells of children living in a region with chipboard industries. These findings, combined with previously reported associations between chipboard industrial activities and respiratory outcomes in children, add to concerns about potential adverse effects of industry-related exposures in the Viadana district.

Citation: Marcon A, Fracasso ME, Marchetti P, Doria D, Girardi P, Guarda L, Pesce G, Pironi V, Ricci P, de Marco R. 2014. Outdoor formaldehyde and NO_2_ exposures and markers of genotoxicity in children living near chipboard industries. Environ Health Perspect 122:639–645; http://dx.doi.org/10.1289/ehp.1307259

## Introduction

Industrial wood manufacturing may be a source of several ambient air pollutants. Mechanical woodworking emits mainly wood dust. In the chipboard production process, urea-formaldehyde resins are commonly used to bond wood particles and laminates together. Both production and use of these bonding agents as well as storing of finished particle boards may release formaldehyde in the atmosphere. A variety of combustion by-products may be generated in wood manufacturing as a consequence of wood waste incineration and heavy traffic, which include nitrogen oxides (NO_x_), carbon monoxide, volatile organic compounds, and, to a minor extent, polycyclic aromatic hydrocarbons, dioxins, and metals (Dahlgren et al. 2003a; de Marco et al. 2010).

**Figure 1:**
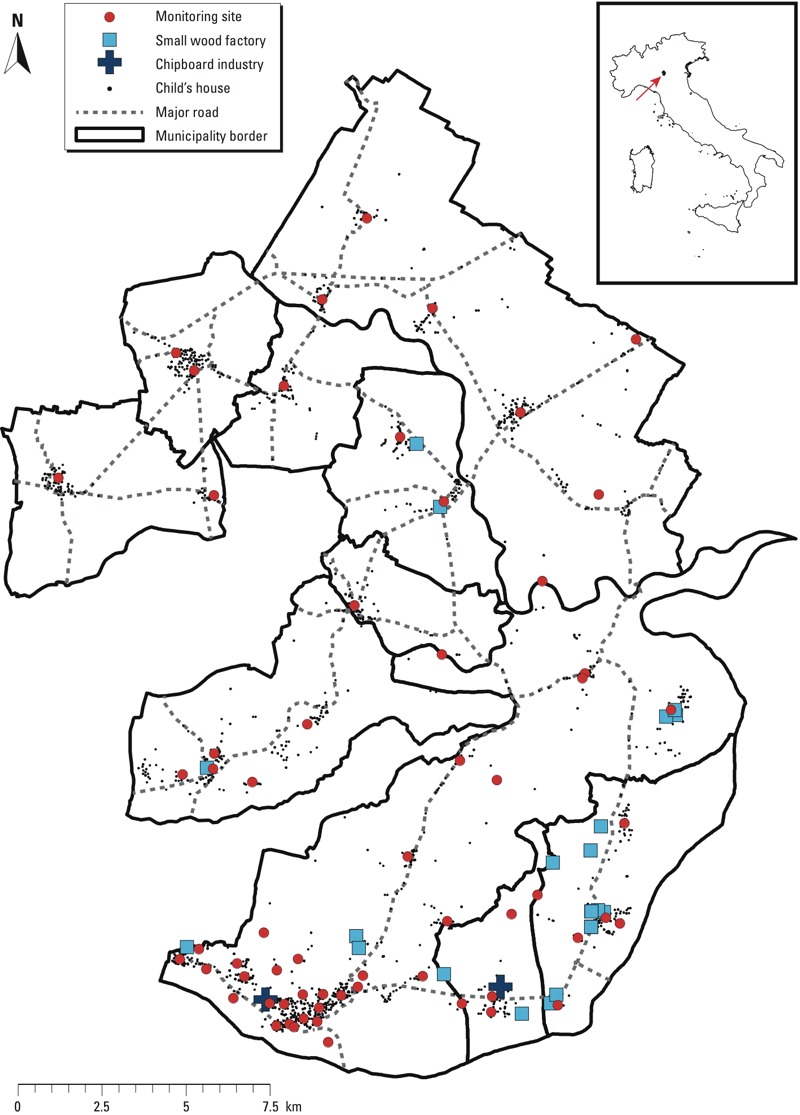
Map of the Viadana district, and small outline map of Italy, with an arrow indicating the location of this district.

Most of the research published on the health effects of pollution from chipboard and wood industries are occupational studies [International Agency for Research on Cancer (IARC) 2006], whereas very few populations exposed through the general environment have been investigated (Dahlgren et al. 2003b; de Marco et al. 2010). The Viadana district is the largest chipboard industrial park in Italy. Two big chipboard industries in the south of the district represent the main industrial emission sources in the area (Figure 1). They include chemical plants for the production of urea-formaldehyde resins, particle board production and storage facilities, and small incinerators. Both industries are under the European Industrial Emissions [Integrated Pollution Prevention Control Directive 2010/75/UE (Italian D.Lgs. 152/06 with subsequent modifications and integrations) (European Union 2010)].

During chipboard panel production, methyl alcohol is used to produce formaldehyde, which is used to synthesize urea-formaldehyde resins that are mixed with wood chips to form particle boards by heat pressing. A small amount of formaldehyde that has not reacted remains in the panel, but most of it is removed by abatement systems. What the abatement systems fail to neutralize contributes to emissions into the atmosphere. Wood waste that is not used in panels is burned to produce steam for internal use.

Smaller wood factories [eight sawmills, five pallet production facilities, six plywood production facilities (two of which include low-volume chipboard production), and three other wood-related activities (furniture production and wood waste storage and recovery facilities)] are spread around the southern and central part of the district. Only a few of these factories burn their own wood waste using small-scale boilers to produce energy for internal use. Therefore, their main emissions are small quantities of dust and combustion gases.

Previous studies by our research team documented that children who lived close to the chipboard industries had more respiratory and irritative symptoms and more frequent hospitalizations for respiratory diseases (de Marco et al. 2010; Girardi et al. 2012; Marchetti et al. 2014; Rava et al. 2011) compared with children living farther away. These analyses, however, were based on distance as a proxy indicator of exposure. As an extension of the previous studies, we modeled outdoor concentrations of formaldehyde and nitrogen dioxide (NO_2_) at the residential addresses of a sample of the previously investigated children, to test whether exposure to these pollutants was associated with evidence of genotoxic damage, assessed by the alkaline comet and micronucleus assays (Dhillon et al. 2004; Fenech et al. 2009) in oral mucosa cells. Both assays have been extensively used in epidemiological studies of chronic occupational and environmental exposures (Neri et al. 2006; Srám et al. 1998; Wilhelm et al. 2007).

## Methods

*Study design*. The Viadana study was a survey on the population of children 3–14 years of age living in the Viadana district, northern Italy, carried out in 2006. At that time, questionnaires were filled in by parents and collected for 3,854 children (93% of the population) (de Marco et al. 2010). During 2010–2011, random samples of the participants in the former study were invited to a new survey (Viadana II). Briefly, children ≥ 12 years of age by December 2010 (*n* = 2,153) were excluded to minimize confounding due to tobacco or alcohol consumption, along with children whose parents answered the English or French versions of the study questionnaire (*n* = 26), and children with missing residential addresses at baseline questionnaire (*n* = 42) (see Supplemental Material, Figure S1). A random sample of 250 children was drawn from each of three strata defined according to the distance from each child’s home (address reported in the baseline questionnaire) to the factories (< 2 km from a chipboard industry, < 4 km from a small wood factory but ≥ 2 km from chipboard industries, ≥ 4 km from any factory). Of these 750 children, 94 were excluded because they had moved outside the district since 2006. The families of the remaining 656 children were invited by mail to participate in the study. Nonresponders were contacted by phone.

The local ethics committee approved the study protocol. The parents (or guardians) of each child signed an informed consent.

*The questionnaire*. The follow-up questionnaire is a short version of the baseline questionnaire on children’s health and risk factors (de Marco et al. 2010), with some additional items on oral hygiene. The address “where the child spends most of his/her time” was collected in both questionnaires (freely available at http://biometria.univr.it/viadanastudy). Children who moved inside the district between the two studies (“movers”) were identified by comparing the addresses reported in the baseline and follow-up questionnaires.

*Collection of cell samples*. After washing out the child’s mouth with tepid water to remove exfoliated dead cells, parents (with/without the assistance of their pharmacist) or local health personnel collected epithelial mucosa cell samples by gently brushing the inside of both cheeks with a cytology brush. The brush was then stirred in a phosphate-buffered saline solution (pH 7.4). Cell suspensions were washed twice with centrifugation at room temperature; then the cells were counted and their viability was determined by the trypan blue exclusion technique.

*Measurement of genotoxic damage*. In the comet assay, cells were processed in alkali conditions and underwent submarine electrophoresis (Faccioni et al. 2003; Fracasso et al. 2009). Ethidium bromide–stained DNA samples were inspected under a fluorescence microscope, and genotoxic damage was quantified using a software-based analysis of electronic images (Comet Assay II; Perceptive Instruments, Bury St. Edmunds, UK). Fifty randomly selected viable cells were examined for each participant. When < 50 cells were available, the number of cells examined was recorded. For each “comet,” DNA damage was quantified as percent of DNA in the tail (tail intensity; TI), tail length (TL; micro-meters), and tail moment (TM) (integrated product of TI and TL, with no measurement unit). The median of each parameter was used as the representative value for each subject (Fracasso et al. 2010).

In the “micronucleus assay,” cell samples were centrifuged in an isotonic buffer to remove oral bacteria. Purified cells were fixed in cold methanol and stained with 4´,6-diamidine-2´-phenylindole dihydrochloride. For each participant 2,000 viable cells were examined using a fluorescence microscope, and the number and frequency (percent) of micronuclei (MN), binucleated cells (BN), and nuclear buds were evaluated according to Tolbert et al. (1991).

The assays were conducted in the laboratory of the Section of Pharmacology, University of Verona, Italy (Faccioni et al. 2003; Fracasso et al. 2009, 2010, 2011).

*Air pollutant monitoring and exposure assignment*. In 2010, ad hoc formaldehyde and NO_2_ measurement campaigns were carried out by radial diffusive passive sampling (Radiello tubes; Fondazione Salvatore Maugeri, Padova, Italy) at 62 sites in the district (Figure 1). Four 1-week campaigns (two in the warm season beginning on 3 June and 29 June, respectively, and two in the cold season beginning on 11 November and 16 December, respectively) were conducted. Half of the sites were selected within a 3-km radius around the chipboard industries, where the highest concentrations of air pollutants were expected. The optimal location of the monitoring sites was chosen using a “partitioning around medoids” algorithm (Kaufman and Rousseeuw 1990), which grouped the points (children’s houses) into equal-sized clusters, and identified the point (medoid) at the minimal distance to all the other points in each cluster. Samplers were then located at these medoids, after having taken special care to select locations that were far away from main streets, crossroads, and point emission sources.

The annual average concentration of formaldehyde and NO_2_ was calculated for each site after adjusting for temporal variation according to the protocol used in the European Study of Cohorts for Air Pollution Effects (ESCAPE) (Cyrys et al. 2012). Specifically, the average concentration of the four campaigns was calculated for the sites with complete data; the difference between the average pollutant concentration for each campaign and this average concentration was subtracted from each measurement to reduce bias due to missing data at sites with fewer than four measurements.

Pollutant annual concentrations at unmeasured locations were interpolated by ordinary kriging (Cressie 1993). The best-fitting models were chosen by minimizing the root mean squared error (RMSE), by leave-one-out cross-validation (LOOCV) (Pebesma and Wesseling 1998). The spatial variogram that provided the best model fit was an exponential class model for both pollutants. The variogram parameters for formaldehyde were partial sill = 0.12, range = 3.5 km, nugget = 0.01, and direction in plane = 90° (east); and the estimated LOOCV-RMSE was 0.089. The variogram parameters for NO_2_ were partial sill = 10, range = 4 km, and direction in plane = 135° (southeast); and the estimated LOOCV-RMSE was 11.997.

The mean annual concentrations estimated at the home addresses reported in the follow-up questionnaire were used as a proxy of the children’s outdoor residential exposure.

Pollutant concentrations are reported in micrograms per cubic meter, where 1 μg/m^3^ = 1.23 ppb at normal temperature (25°C) and pressure (103.5 kPa) (IARC 2006).

*Statistical analysis*. Data were summarized with means ± SD, medians (1st and 3rd quartiles), and percentages, as appropriate. Comparisons across groups were performed by analysis of variance for quantitative variables and Pearson’s chi-square test for categorical variables. The associations between exposure to formaldehyde or NO_2_ (standardized) and markers of genotoxic damage were evaluated using linear regression models for the normally distributed markers (TI, TL, TM, and BN), and expressed as linear regression coefficients. In the case of the skewed markers (MN and bud counts), negative binomial regression models were used because of overdispersion, and the associations were expressed as relative risks (RRs). Analyses of the comet assay markers were weighted for the number of cells examined per subject. Models were adjusted for children’s sex, age (continuous in years), nationality (Italian or foreign if neither parent was Italian), parents’ education (maximum among parents, coded as primary school or less, secondary/professional school high school, university), parents’ smoking habits (neither smoked, one or both smoked), exposure to tobacco smoking at home (none or any), average time of air refreshing (keeping windows open for < 15 min/day, about 30 min/day, ≥ 1 hr/day), questionnaire-reported traffic near home (high if cars or trucks passed the child’s house frequently/constantly; low if cars and trucks never/seldom passed), presence of orthodontic appliance, DMFT (decayed, missing, filled teeth) score (≥ 1 vs. 0) (Nishi et al. 2002) and person who collected the cell sample (parents alone, parents assisted by pharmacist, health personnel). In sensitivity analyses, *a*) three more indicators of indoor air quality, derived from the baseline questionnaire, were added [age of the house (≤ 5 or > 5 years), age of nonwooden furniture in the child’s bedroom (none, < 3 years, ≥ 3 years), and presence of double-glazed windows in children’s bedroom (yes or no)] to the main model; because these indicators referred to the house inhabited at baseline, these analyses were performed after excluding the movers (*n* = 59); *b*) the analyses were restricted to the children who lived at < 4 km from the two chipboard industries (*n* = 172).

Statistical significance was set at the 5% level. The statistical analysis was performed using STATA software, release 12.1 (StataCorp, College Station, TX, USA) and R version 3.0 (http://www.r-project.org).

## Results

*Participation rates and children’s characteristics*. Overall, 413 (63%) children participated in the Viadana II study. Participation ranged from 61 to 66% for the three sampling strata (see Supplemental Material, Table S1). Participating children were less likely to be foreigners and to have smoking parents (*p* < 0.001) than nonparticipating children (*n* = 243) (see Supplemental Material, Table S2).

Forty-four percent of the participants were female, and they were 9.4 ± 1.6 years of age on average. The children from the three strata were similar with regard to sex and age, parents’ education and nationality, and exposure to passive smoking (Table 1). The children who lived closer to the chipboard and wood factories reported a greater exposure to vehicular traffic.

**Table 1:** Characteristics of the children by distance of their houses to the wood factories [*n* (%)].

Characteristic	≥ 4 km from any wood factory (*n* = 134)	< 4 km from a small wood factory (*n* = 151)	< 2 km from a chipboard industry (*n* = 128)	*p*-Value
Female sex	58 (43.3)	62 (41.1)	62 (48.4)	0.45
Age (years)^*a*^	9.2 ± 1.7	9.6 ± 1.5	9.5 ± 1.6	0.06
Foreign nationality	11 (8.3)	17 (11.4)	9 (7.1)	0.43
Parents’ education				0.44
Primary school or less	3 (2.3)	6 (4.1)	2 (1.6)
Secondary/professional school	42 (31.8)	38 (25.7)	32 (25.6)
High school	63 (47.7)	85 (57.4)	68 (54.4)
University	24 (18.2)	19 (12.8)	23 (18.4)
Smoking parents	34 (25.6)	39 (27.7)	44 (33.9)	0.31
Exposure to tobacco smoking at home	10 (7.6)	21 (14.1)	15 (11.7)	0.23
High residential traffic level	74 (55.6)	82 (55.4)	88 (68.8)	0.04
Orthodontic appliance	25 (18.8)	33 (22.5)	22 (17.5)	0.56
DMFT score ≥ 1	62 (46.3)	64 (42.4)	60 (46.9)	0.71
Person who collected the cell sample				0.40
Parents, unassisted	23 (17.2)	16 (10.6)	19 (14.8)
Parents, assisted	85 (63.4)	95 (62.9)	82 (64.1)
Health personnel	26 (19.4)	40 (26.5)	27 (21.1)
Average time of air refreshing				0.63
< 15 min/day	31 (23.5)	34 (22.8)	34 (27.2)
About 30 min/day	36 (27.3)	45 (30.2)	41 (32.8)
≥ 1 hr/day	65 (49.2)	70 (47.0)	50 (40.0)
Age of the house ≤ 5 years^*b*^	11 (11.0)	19 (17.9)	15 (16.9)	0.34
Nonsolid (chipboard, plywood) wooden furniture in child’s bedroom^*b*^				0.20
None	31 (26.7)	28 (23.0)	17 (16.5)
< 3-year-old furniture	40 (34.5)	33 (27.1)	37 (35.9)
≥ 3-year-old furniture	45 (38.8)	61 (50.0)	49 (47.6)
Double-glazed windows in child’s bedroom^*b*^	73 (62.9)	78 (64.5)	74 (70.5)	0.46
The distance to the closest wood/chipboard factory was calculated from the home address reported in the follow-up questionnaire (2010–2011). ^***a***^Age in December 2010; mean ± SD reported. ^***b***^This information was collected in the baseline questionnaire only; thus, 59 children that had moved since 2006 were excluded.				

*Spatial distribution of air pollutants*. After adjusting for temporal variability, the estimated annual average concentrations of formaldehyde and NO_2_ were 2.5 ± 0.3 μg/m^3^ and 16.0 ± 3.5 μg/m^3^, respectively (see Supplemental Material, Table S3). Formaldehyde concentrations were higher in the warm season than in the cold season (mean ± SD, 2.8 ± 0.4 and 2.1 ± 0.5 μg/m^3^ respectively, *p* < 0.001), whereas NO_2_ concentrations showed an opposite trend (12.9 ± 3.4 vs. 18.8 ± 5.6 μg/m^3^, *p* < 0.001).

**Figure 2:**
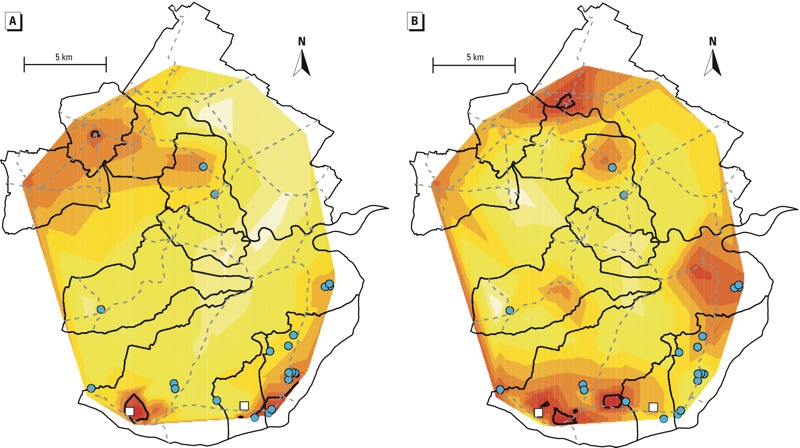
Map of the estimated annual average concentrations of formaldehyde (A) and NO2 (B). The white to red graduation indicates lower to higher concentrations. Dashed gray lines represent major roads. Black contour lines delimit the area where the concentration estimates were above the 90th percentile of the distribution (hot spots). The chipboard and other wood factories are represented by white squares and blue circles, respectively.

Formaldehyde modeled concentrations were higher in the south than in the rest of the district (Figure 2A). In particular, the widest hot spot (concentrations > 90th percentile) was estimated around the chipboard industry in the southwestern area. NO_2_ modeled concentrations showed a slightly greater spatial variability (Figure 2B): Hot spots were located mostly in the south of the district, but one hot spot was located in the north.

The children who lived close to the chipboard industries were the most exposed to both pollutants (*p* < 0.001). In this group, the mean concentrations of formaldehyde and NO_2_ were 2.6 ± 0.2 and 17.5 ± 1.7 μg/m^3^ respectively, whereas they were 2.4 ± 0.2 and 15.4 ± 1.9 μg/m^3^, respectively, for children living close to the small factories, and 2.5 ± 0.2 and 15.2 ± 2.0 μg/m^3^, respectively, for children living far from the factories (see Supplemental Material, Figure S2).

*Formaldehyde and NO_2_ exposure and genotoxic response*. The distribution of the markers of genotoxicity is described in Table 2. In the adjusted analyses (Table 3), 1-SD increases in formaldehyde (0.20 μg/m^3^) and NO_2_ (2.13 μg/m^3^) were associated with 12% (RR = 1.12; 95% CI: 1.02, 1.23, *p* = 0.023) and 16% (RR = 1.16; 95% CI: 1.06, 1.26, *p* = 0.001) relative increases in average numbers of nuclear buds, respectively. A 1-SD increase in formaldehyde was associated with a 0.13% (95% CI: 0.03, 0.22%) higher TI (*p* = 0.012) and a 0.007 (95% CI: 0.001, 0.012) higher TM. A 1-SD increase in NO_2_ was associated with a 0.13% (95% CI: 0.07, 0.19%) increase in BN frequency (*p* < 0.001). Associations of the other outcomes with the exposures were not statistically significant.

**Table 2:** Distribution of the markers of genotoxic damage in the children.

Outcome	Mean ± SD	CV	Median	1st, 3rd quartile	Min, max
Comet assay^*a*^ (*n* = 340)
Tail intensity (%)	3.25 ± 0.88	0.27	3.20	2.69, 3.76	0.63, 6.85
Tail length (μm)	11.69 ± 2.11	0.18	11.60	10.36, 12.83	5.92, 19.24
Tail moment	0.20 ± 0.05	0.25	0.20	0.17, 0.23	0.08, 0.40
Micronuclei assay (*n *= 411)
Binucleated cells (%)	1.83 ± 0.64	0.35	1.80	1.40, 2.20	0.60, 4.20
Micronuclei (%)	0.12 ± 0.09	0.71	0.10	0.10, 0.20	0.00, 0.40
Nuclear buds (%)	0.23 ± 0.23	1.01	0.20	0.10, 0.30	0.00, 2.40
Abbreviations: max, maximum; min, minimum. ^***a***^Weighted for the number of cells examined (50 when available).					

**Table 3:** Estimated associations (95% CI) of exposure to formaldehyde and NO_2_ with markers of genotoxic damage.

Outcome	Formaldehyde (0.20 μg/m^3^)^*a*^		NO_2_ (2.13 μg/m^3^)^*a*^	
	Crude	Adjusted^*b*^	Crude	Adjusted^*b*^
Comet assay^*c*^
No. of subjects with complete information	336	310	336	310
Tail intensity (% change)	0.10 (0.00, 0.19)*	0.13 (0.03, 0.22)*	0.04 (–0.06, 0.13)	0.06 (–0.05, 0.16)
Tail length (μm change)	–0.05 (–0.27, 0.18)	–0.06 (–0.29, 0.17)	0.08 (–0.15, 0.32)	0.10 (–0.14, 0.34)
Tail moment	0.003 (–0.002, 0.008)	0.007 (0.001, 0.012)*	0.002 (–0.004, 0.008)	0.004 (–0.002, 0.010)
Micronucleus assay
No. of subjects with complete information	406	374	406	374
Binucleated cells (% change)	0.02 (–0.04, 0.09)	0.02 (–0.05, 0.08)	0.13 (0.07, 0.20)^#^	0.13 (0.07, 0.19)^#^
Micronuclei (RR)	0.99 (0.93, 1.06)	0.98 (0.91, 1.06)	1.01 (0.94, 1.08)	1.00 (0.93, 1.07)
Nuclear buds (RR)	1.13 (1.03, 1.25)*	1.12 (1.02, 1.23)*	1.18 (1.09, 1.28)^#^	1.16 (1.06, 1.26)**
^***a***^Corresponding to a 1-SD increase in exposure. ^***b***^Adjusted for sex, age, nationality, parents’ education and smoking habits, exposure to tobacco smoking at home, average time of air refreshing, residential traffic level, presence of orthodontic appliance, DMFT score, person who collected the cell sample. ^***c***^Weighted for the number of cells examined (50 when available).*p < 0.05. **p < 0.01. ^#^*p *< 0.001.				

Compared with the main analyses, adjustment for additional indicators of indoor air quality provided very similar association estimates (see Supplemental Material, Table S4).

The analyses on subsets of children living in a 4-km radius from the chipboard industries resulted in stronger estimates for the associations that were statistically significant in the main analyses (see Supplemental Material, Table S4). An exception is the association between formaldehyde and nuclear buds, which shifted to null.

## Discussion

Previous studies documented that the children who lived closer to the big chipboard industries in the south of the Viadana district had more asthma-like symptoms, irritative symptoms of the eyes and airways, and hospitalizations for respiratory diseases than did children who lived farther away, whereas skin-related disorders were not in excess (de Marco et al. 2010; Girardi et al. 2012; Marchetti et al. 2014; Rava et al. 2011). These findings were compatible with exposure of the mucosa and target organs to irritating chemicals emitted from anthropogenic emission sources. The present investigation adds to this evidence by documenting statistically significant associations between estimated residential outdoor levels of formaldehyde and NO_2_ and markers of genotoxic responses in oral mucosa cells.

Exposure to air pollutants was estimated by monitoring, followed by exposure modelling, which is usually done when individual measurements are not feasible (Jerrett et al. 2005). The number of monitoring sites was in the range suggested by others (Hoek et al. 2008), and the sites were placed using a statistical algorithm (Kaufman and Rousseeuw 1990) that yielded the optimal location when taking into account both the density of the sampling population and the level/variability of pollution. The Viadana district is a flat and relatively wind-free area (average wind speed in the last years, 2.3 m/sec) (Marchetti et al. 2014), characterized by a mild continental climate, and it is not affected by sudden changes in meteorological conditions. Thus, orographic or extreme climatic factors are unlikely to have biased exposure assignment. Annual average concentrations of formaldehyde and NO_2_ were used as markers of chronic exposure to human polluting activities in the study area. Both chemicals are relatively cheap and simple to measure by passive sampling, they have a low regional background contribution, and their concentrations are mostly influenced by local sources (Cyrys et al. 2012).

*Exposure to formaldehyde*. Knowledge of the health effects of formaldehyde mainly comes from studies of indoor and occupational exposures (IARC 2006; McGwin et al. 2010), or controlled-exposure experimental studies (Lang et al. 2008). Formaldehyde can cause eye and respiratory tract irritation at low levels by trigeminal stimulation (Arts et al. 2006). This leads to reflex responses such as lacrimation, coughing, sneezing, or rhinorrhea. Formaldehyde exposure is suspected to be causally linked to asthma in children (McGwin et al. 2010). There is evidence that it causes nasopharyngeal cancer and leukemia in humans (IARC 2006). Formaldehyde can cross-link DNA and proteins in cells, which is considered to be its primary genotoxic effect. Substantial experimental data suggest that the dose–response relationship for health effects of formaldehyde is nonlinear (Nielsen and Wolkoff 2010).

Outdoor levels of formaldehyde in remote areas are low, generally < 1 μg/m^3^ (IARC 2006). In populated environments, formaldehyde is emitted by incomplete combustion of hydrocarbon fuels and formed secondarily by hydrocarbon photooxidation. Urban concentrations usually range between 1 and 20 μg/m^3^, although heavy traffic and episodes of severe inversions can lead to much higher concentrations (IARC 2006; Kheirbek et al. 2012).

The average concentration measured in the Viadana district was 2.5 ± 0.3 μg/m^3^. Apart from the industrial settlements in the south, the Viadana district can be considered a rural area. No highways cross the district, nor are there high-traffic areas or industrial plants other than the wood and chipboard factories.

The exposure map of formaldehyde (Figure 2A) shows that the highest concentrations were estimated in the south, especially around the southwestern area of the chipboard industry. Accordingly, the exposure estimates assigned to the children who lived at < 2 km from the chipboard industries were higher than for the children who lived farther away.

*Exposure to NO_x_*_._ NO_x_ are produced by combustion at high temperature. Nitrogen oxide is rapidly oxidized to NO_2_ in the atmosphere. NO_2_ is an important intermediate for the production of toxic secondary pollutants, including ozone, nitric and nitrous acids, and alkyl nitrates (Baldacci et al. 2009). NO_x_ have been associated with several short- and long-term adverse health effects including mortality, hospitalizations, and COPD (chronic obstructive pulmonary disease) in adults, asthma and wheezing in children (Brauer et al. 2007; Schikowski et al. 2014).

The main sources of NO_x_ in the study area were wood waste incineration and power generation in the factories, vehicular traffic, and domestic heating. The exposure map of NO_2_ (Figure 2B) shows that the top 10% concentration hot spots were mainly in the south, and NO_2_ exposure was higher close to the chipboard industries than farther away. This suggests that the direct or indirect contribution of the chipboard industries to the ambient concentrations of NO_2_ in the district was not negligible.

The average annual NO_2_ concentration measured was 16.0 ± 3.5 μg/m^3^. This is similar to the concentrations measured in some less polluted cities across Europe in ESCAPE, where median annual concentrations ranged between 14 and 19 μg/m^3^ (Cyrys et al. 2012). During the same year (2010), the concentration of NO_2_ measured by the routine monitoring stations located in the closest town, Mantua [49,328 inhabitants in December 2011, according to the Italian national Institute of Statistics (ISTAT), Rome Italy], ranged between 30 and 35 μg/m^3^ (ARPA Lombardia 2010). The difference in NO_2_ concentrations between these two areas may be attributable to differences in traffic intensity and population/building density, but it also may be partly attributable to the different measurement methodology used (chemiluminescence monitors) (Cyrys et al. 2012).

*Exposure to air pollution and markers of genotoxicity*. Despite the increasing interest in recent years on using exfoliated oral mucosa cells (Holland et al. 2011), at present there are no reference values for the comet and micronucleus assays on this cell type. In published studies, large variability is observed according to population’s age, exposures, lifestyles, and disease status (Faccioni et al. 2003; Sisenando et al. 2012; Thomas et al. 2008). As expected, we found smaller mean values of the comet assay markers in our study than we had previously reported in young adults exposed to smoking, alcohol, and metal release from orthodontic appliances (Faccioni et al. 2003). In fact aging and exposure to pollutants are known to be associated with increased genotoxicity (Holland et al. 2011).

DNA repair efficiency can be jeopardized by several factors, including a reduced capacity to recognize DNA damage, repair enzyme deficiency, and depletion of the antioxidant cellular capacity as a consequence of acute or chronic exposure to oxidative stress. The comet assay detects DNA single-strand breaks, alkali-labile sites, and incomplete excision repair in proliferating and nonproliferating cells, representing transient promutagenic lesions (Collins 2009). When inadequate DNA repair occurs, fixed mutations can be produced, which can be detected by cytogenetic tests such as the micronucleus assay (Tolbert et al. 1991). The mechanisms leading to the formation of micronuclei are chromosome breakage and incorrect chromosome-segregation system. Both mechanisms require mitotic or meiotic cell division.

We found that a 1-SD increase in estimated exposure to formaldehyde (0.20 μg/m^3^) was significantly associated with higher TI (0.13%; 95% CI: 0.03, 0.22%) and TM (0.007; 95% CI: 0.001, 0.012), as well as with an increased frequency of nuclear buds (RR = 1.12; 95% CI: 1.02, 1.23). An association between formaldehyde exposure and evidence of genotoxic damage has been reported *in vitro* (Schmid et al. 2007) and in occupational studies (Costa et al. 2011).

Exposure to NO_2_ (2.13 μg/m^3^) was significantly associated with the frequency of BN (0.13%; 95% CI: 0.07, 0.19%) and nuclear buds (RR 1.16; 95% CI: 1.06, 1.26). Other studies on the relationship between NO_2_ and markers of genotoxicity showed contrasting findings (Koehler et al. 2010; Srám et al. 1998).

A comparison of our results with those of other studies is hindered by the paucity of publications that studied genotoxicity in oral mucosa cells, the heterogeneity of populations and exposures studied, as well as the lack of technical standards for these assays (Holland et al. 2008). An evaluation of the magnitude of the estimated associations is therefore difficult, also considering that the relationship between genotoxic damage in oral mucosa and health outcomes is not completely clear. To our knowledge, there are no published articles that studied the relationship of environmental exposures with BN and nuclear buds in oral mucosa cells. As regards the comet assay, the magnitude of our association estimates is in line with our study on young adults exposed to nickel and cobalt release from orthodontic appliances (Faccioni et al. 2003), although the population investigated is very different from that investigated in the present study.

Except for the association between formaldehyde and nuclear buds, which shifted from positive to null, positive associations observed in the population as a whole were stronger in magnitude when analyses were restricted to study participants living < 4 km from the chipboard industries. This is consistent with expectations for a causal role of industry-related exposures if one assumes that exposure classification was more accurate and/or more specific to industry-related exposures in the subpopulation living closer to the industries than in the population as a whole.

The indoor environment is a major contributor of several air pollutants, including formaldehyde (Daily et al. 1981). One limitation of this study is that we did not have indoor air pollution measurements to adjust for, in order to rule out the confounding by indoor exposures. However, the analyses were adjusted for some indicators of indoor air quality (passive smoking, time of air refreshing, traffic near home, socioeconomic factors). Moreover, when three other indicators of indoor air quality were included, the results were confirmed (see Supplemental Material, Tables S4), although we acknowledge that these analyses adjusting for relatively rough indicators do not rule out confounding by indoor exposures. Finally, there was no confounding due to tobacco or alcohol consumption or occupational exposures, because all the children were < 12 years old.

Cell processing in the comet assay may result in inadequate samples that contain insufficient viable cells. In contrast, the cells do not undergo any treatment in the micronucleus assay. Because of these technical differences, a lower number of children were successfully tested by the comet assay (*n* = 340) than by the micronucleus assay (*n* = 411), and this resulted in a lower statistical power for the former.

It could be argued that the small spatial exposure contrasts observed cannot cause harmful effects on health. However, our exposure measurements should be interpreted in the light of the following considerations. First, passive sampling gives no information on variability of pollutant concentrations over the sampling period. Thus, the children exposed to higher “average” concentrations may also have been more exposed to pollutant “peaks.” Annual average exposure estimates were based on only four 1-week sampling periods, which may have led to exposure misclassification over the entire year. Second, the monitored pollutants were used as “markers” of human polluting activities: Higher concentrations may indicate greater exposure to a mixture of other unmeasured pollutants. Third, as previously mentioned, an under-estimation of actual levels of pollution could have occurred as a consequence of the measurement methodology used (Cyrys et al. 2012), but spatial contrasts in exposure are more important than real levels in our study. Finally, industrial production in the district was lower at the time of the study than in previous decades as a consequence of the recent economic crisis. Our findings could therefore have been blunted with respect to periods of full industrial activity.

## Conclusions

The main strength of this study is that both exposures and the outcomes were based on objective measurements. Markers of early cell damage were available on an individual basis. The association between exposures and the outcomes was assessed in a representative sample of children. The limitations are that personal and indoor exposure measurements were not available, and that the participation rate was only moderate. Exposure in a time window closer to the time of outcome assessment might have been more relevant than annual average exposure for genotoxic responses. However, this possible exposure misclassification may have resulted in an underestimation of the associations rather than in an overestimation (Seixas and Sheppard 1996).

In conclusion, our exposure estimates suggest that the children living close to the chipboard industries in the Viadana district were more exposed to air pollutants potentially related to the industrial activities than were children living farther away. On average, children exposed to higher pollutant concentrations had higher levels of markers of genotoxic responses in oral mucosa cells than did less-exposed children. Our findings support that chronic exposure to low air pollution levels may determine early cell damage. Oral mucosa cells are exfoliated cells characterised by very rapid processes of elimination, and protective mechanisms of DNA repair exist. Nonetheless, chronic exposure to pollution may result in decreased host defenses, especially in children, and genome damage that occurs at a young age may influence the lifetime risk of delayed health effects, including cancer (Bonassi et al. 2011; Poirier 2012).

The present findings add to concerns about the potential for adverse health effects of industry-related exposures among children living in the Viadana district. Primary prevention to reduce air pollution in the area and a follow-up of the paediatric population are recommended.

## Supplemental Material

(600 KB) PDF
