# Supplementary material for: Outdoor Formaldehyde and NO2 Exposures and Markers of Genotoxicity in Children Living Near Chipboard Industries
Source: Environ Health Perspect. 2014 Apr 2;122(6):639–45. doi: 10.1289/ehp.1307259 (PMC4050513; doi:10.1289/ehp.1307259)
Supplement: (600 KB) PDF [file ehp.1307259.s001.pdf]

Supplemental Material

Outdoor Formaldehyde and NO_2 Exposures and Markers of Genotoxicity in Children Living Near Chipboard Industries

Alessandro Marcon, Maria Enrica Fracasso, Pierpaolo Marchetti, Denise Doria, Paolo Girardi, Linda Guarda, Giancarlo Pesce, Vanda Pironi, Paolo Ricci, and Roberto de Marco

Table of Contents **Page**

Table S1. Participation rates and number (%) of genotoxicity assays carried out for each sampling stratum, defined according to the distance of the children's houses (addresses collected by the baseline questionnaire) to the factories. 2

Table S2: Main characteristics of the children who did and did not participate in the study. 3

Table S3: Descriptive statistics on formaldehyde and NO_2 concentrations at the four 1-week measurement campaigns, and annual average concentrations. 4

Table S4. Estimated associations (95% CI) of exposure to formaldehyde and NO_2 with markers of genotoxic damage: comparison between the main and sensitivity analyses. 5

Figure S1: Selection of the children and participation in the Viadana II study. 6

Figure S2: Box-plots representing the distribution of modelled exposure to formaldehyde and NO_2 by distance of children's houses to the factories. 7

Table S1. Participation rates and number (%) of genotoxicity assays carried out for each sampling stratum, defined according to the distance of the children's houses (addresses collected by the baseline questionnaire) to the factories.

**Participation rates**	**≥ 4 km from any wood factory (n=205)**	**<4 km from a small wood factory and ≥2 km from the chipboard industries (n=236)**	**<2 km from a chipboard industry (n=215)**
N. children participating[a]	135 (66%)	146 (62%)	132 (61%)
Micronuclei assays	135 (66%)	144 (61%)	132 (61%)
Comet assays	122 (60%)	115 (49%)	103 (48%)

[a]Participation was defined as having the questionnaire answered and one (or both) the genotoxicity assays carried out.

Table S2: Main characteristics[a] of the children who did and did not participate in the study.

**Characteristics**	**Participants[c] (n=413)**	**Non participants (n=243)**	**p-value**
Female sex	182 (44.0)	107 (44.1)	0.999
Age (y)[b]	5.5±0.1	5.6±0.1	0.408
Foreign nationality	37 (9.0)	43 (18.1)	0.001
Parents' education			0.207
Primary school or less	11 (2.7)	10 (4.3)	
Secondary or professional	112 (27.6)	75 (32.3)	
High school	216 (53.3)	120 (51.7)	
University	66 (16.3)	27 (11.6)	
Smoking parents	142 (35.8)	112 (48.9)	0.001
Exposure to tobacco smoke at home	64 (15.7)	47 (20.1)	0.179
High residential traffic level	242 (59.2)	152 (64.7)	0.179

[a]Information was obtained from the baseline questionnaire. N (%) reported, unless stated otherwise. [b]Age in December 2006; mean ± SD reported. [c]Participation was defined as having the questionnaire answered and one (or both) the genotoxicity assays carried out.

Table S3: Descriptive statistics on formaldehyde and NO_2 concentrations at the four 1-week measurement campaigns, and annual average concentrations.

**Statistics**	**Week 1**	**Week 2**	**Week 3**	**Week 4**	**Average**[a]
Starting date	03/06/2010	29/06/2010	11/11/2010	16/12/2010	
Formaldehyde, mean ± SD (µg/m^3)	2.3±0.5	3.4±0.7	1.6±0.4	2.6±0.6	2.5±0.3
Formaldehyde, coefficient of variation	0.21	0.22	0.26	0.24	0.14
NO_2, Mean ± SD (µg/m^3)	12.1±4.5	13.9±4.2	14.9±5.8	22.8±9.2	16.0±3.5
NO_2, coefficient of variation	0.37	0.30	0.39	0.40	0.22

[a]Adjusted for temporal variation to account for missing data (at monitoring sites with <4 measurements).

Table S4. Estimated associations (95% CI) of exposure to formaldehyde and NO_2 with markers of genotoxic damage: comparison between the main and sensitivity analyses.[a]

Outcome	Formaldehyde: main analysis	Formaldehyde: distance to chipboard industries < 4 km	Formaldehyde: additional adjustment for proxies of indoor air quality[c]	NO_2: main analysis	NO_2: distance to chipboard industries < 4 km	NO_2: additional adjustment for proxies of indoor air quality[c]
**Comet assay**[b]						
number of subjects with complete information	310	118	229	310	118	229
Tail intensity (TI): % change	0.13 (0.03, 0.22)*	0.19 (0.04, 0.34)*	0.12 (0.01, 0.23)*	0.06 (-0.05, 0.16)	0.03 (-0.15, 0.21)	0.09 (-0.03, 0.21)
Tail length (TL): µm change	-0.06 (-0.29, 0.17)	-0.10 (-0.46, 0.26)	-0.01 (-0.27, 0.25)	0.10 (-0.14, 0.34)	0.24 (-0.16, 0.64)	0.04 (-0.24, 0.31)
Tail moment (TM)	0.007 (0.001, 0.012)*	0.012 (0.003, 0.020)**	0.007 (0.001, 0.014)*	0.004 (-0.002, 0.010)	0.006 (-0.004, 0.016)	0.005 (-0.002, 0.012)
**Micronucleus assay**						
number of subjects with complete information	374	155	273	374	155	273
Binucleated cells (BN): % change	0.02 (-0.05, 0.08)	-0.08 (-0.20, 0.03)	0.02 (-0.05, 0.10)	0.13 (0.07, 0.19)***	0.19 (0.07, 0.31)**	0.15 (0.07, 0.22)***
Micronuclei (MN): RR	0.98 (0.91, 1.06)	0.93 (0.82, 1.05)	0.97 (0.89, 1.06)	1.00 (0.93, 1.07)	1.05 (0.92, 1.21)	0.99 (0.91, 1.08)
Nuclear buds: RR	1.12 (1.02, 1.23)*	1.02 (0.89, 1.18)	1.11 (1.01, 1.23)*	1.16 (1.06, 1.26)**	1.23 (1.06, 1.41)**	1.17 (1.07, 1.28)**

*p<0.05, **p<0.01, ***p<0.001

[a]Both the main and sensitivity analyses were adjusted for sex, age, nationality, parents' education and smoking habits, exposure to tobacco smoke at home, average time of air refreshing, residential traffic level, presence of orthodontic appliance, DMFT score, person who collected the cell sample. Estimates are given for a 1-SD increase in exposure (0.20 $\mu g/m^3$ and 2.13 $\mu g/m^3$ for formaldehyde for NO_2, respectively). [b]Weighted for the number of cells examined (50 when available). [c]Also adjusted for age of the house ≤5 years, non-solid (chipboard, plywood) wooden furniture in child's bedroom, double glazed windows in child's bedroom (information retrieved from the baseline questionnaire)

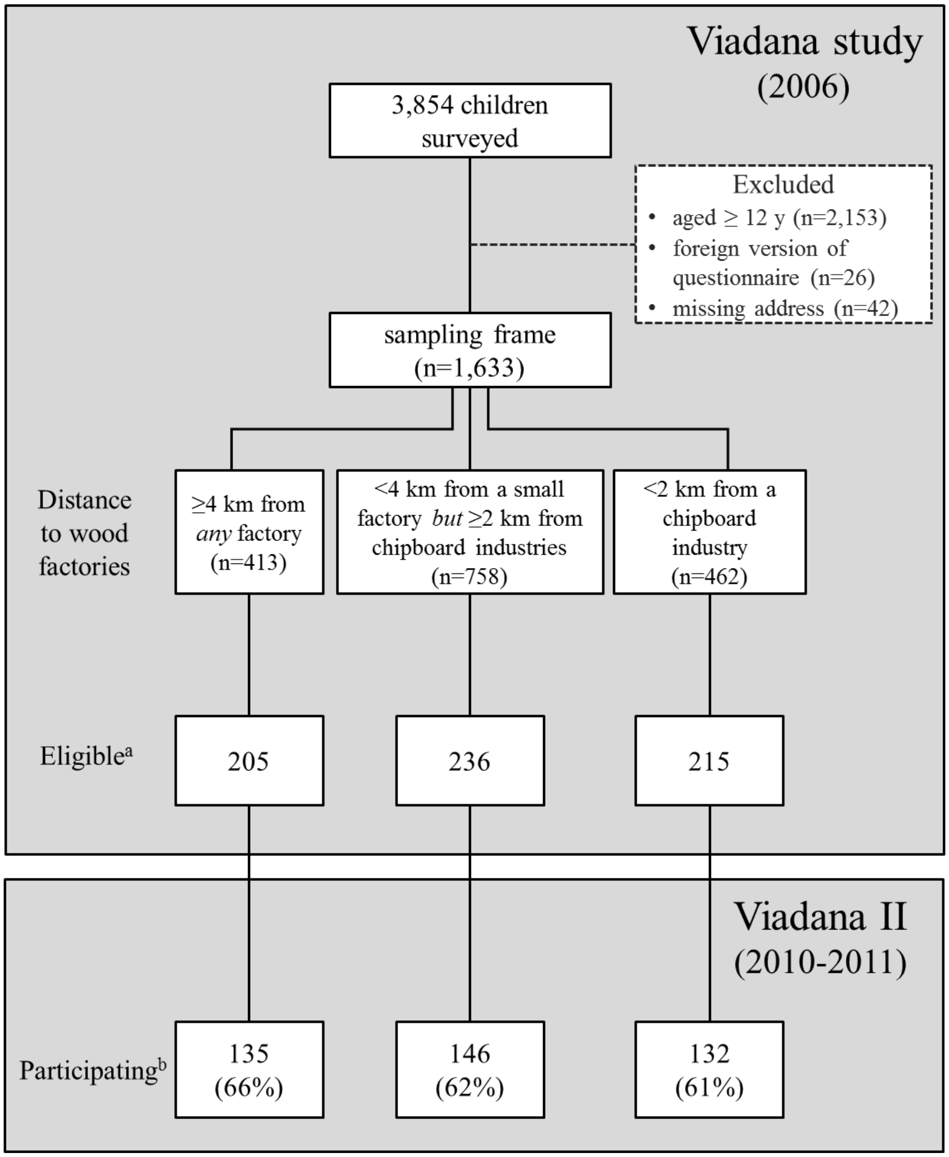

Figure S1: Selection of the children and participation in the Viadana II study. [a]250 children were randomly sampled from each sampling stratum. Then the children who had moved outside the Viadana district between 2006 and 2010 were excluded (n=94 in total). [b]Participation was defined as having the questionnaire answered and one (or both) the genotoxicity assays carried out.

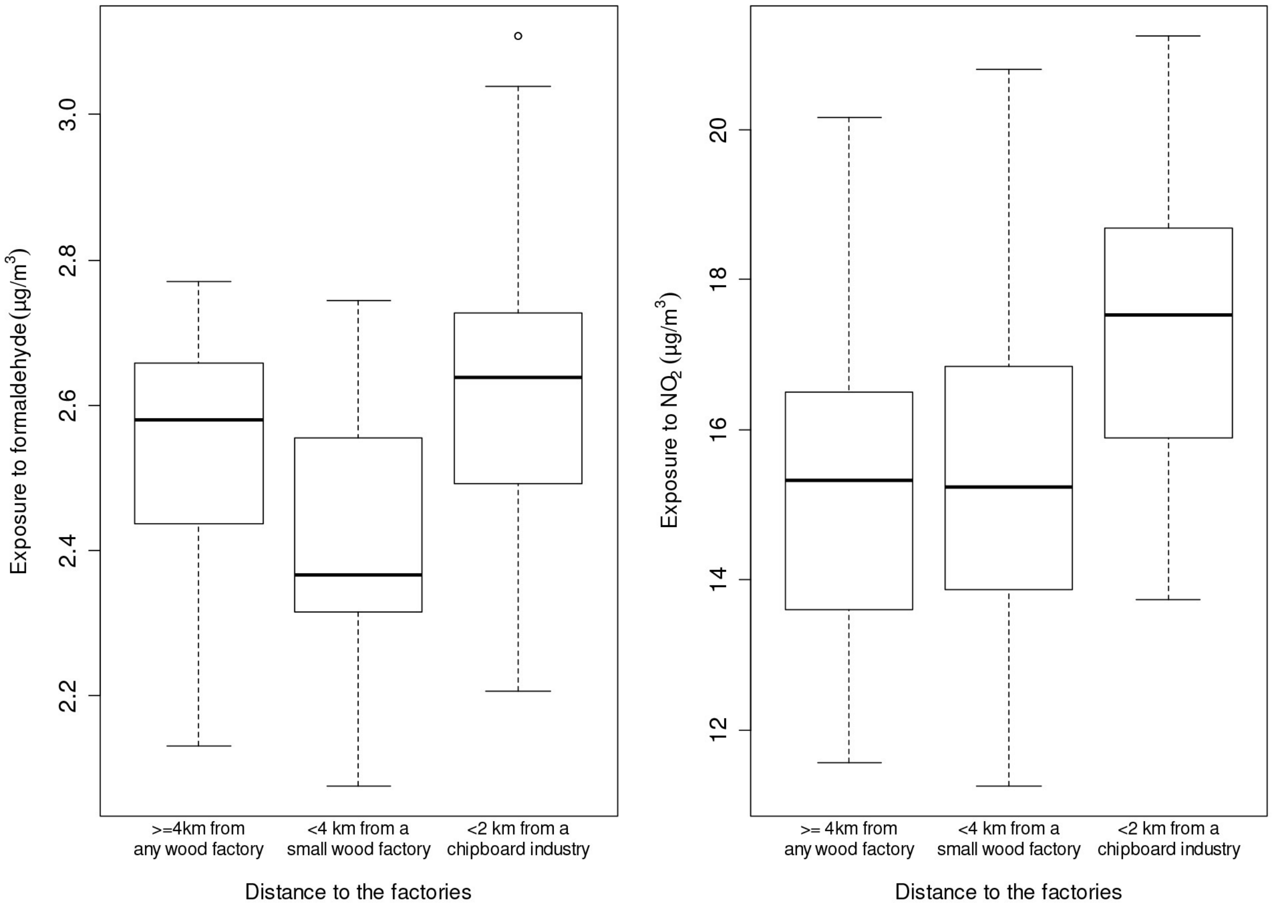

Figure S2: Box-plots representing the distribution of modelled exposure to formaldehyde and NO_2 by distance of children's houses to the factories.[a]

[a]Boxes extend from the 25th to the 75th percentile, horizontal bars represent the median, whiskers extend 1.5 times the length of the interquartile range (IQR) above and below the 75th and 25th percentiles, respectively, and outliers are represented as points.
